# Our New York Letter

**Published:** 1887-02

**Authors:** 


### Correspondence.

### OUR NEW YORK LETTER.

MEASLES IN NEW YORK—DIPHTHERIA AND ITS TREATMENT BY INTUBATION OF THE LARYNX—A DOCTOR PROSCRIBED FOR DOING HIS DUTY—FREE NIGHT SERVICE FOR THE POOR OF NEW YORK—A NEW HOSPITAL FOR CONSUMPTIVES, ETC.

*Editors Atlanta Medical and Surgical Journal:*

Measles heads the list of contagious diseases in New York just now, and the number of cases reported is very large, although the disease cannot be said to be truly epidemic in any particular part of the city, for it exists with about equal prevalence throughout the tenement districts. Nearly all our newspapers are publishing startling accounts of its rapid spread throughout the densely populated portions of the city and the great mortality among its victims. The excitement attendant upon all this undoubtedly causes many physicians to obey the law and report their cases of measles, who otherwise would never think of paying any attention to the subject. Thus it is that a larger number of cases are reported, giving the public a somewhat exaggerated idea of its alarming prevalence this season as compared with others; but it is really true that there is an unusual number of cases in New York and many of them appear to be of a malignant type. Adults seem to escape the contagion less frequently than usual. The mortality has been highest among children under two years of age. During the first eleven days of December, 1886, the deaths from measles had run up to 108, and this was 102 more than occurred through the entire month of December, 1885. For the week ending January 1st, 1887, there were 468 cases reported and sixty-three deaths, and since then the records have shown no diminution in the progress of this disease.

Next in prevalence among the contagious diseases comes diphtheria. In this disease intubation of the larynx, according to Dr. Joseph O'Dwyer's method, has been frequently practiced during the present fall and winter. Intubation, though still in its infancy, is growing rapidly in popularity and taking the place of tracheotomy with many of our practitioners. Quite a number of physicians here are experts in intubation and their services are in considerable demand by the doctors inexperienced in this procedure. Others are rapidly acquiring the necessary skill and the attention of the profession is being more and more called to the subject by papers read before our societies. The idea is growing in favor continually and the number of its opponents is small. A complete outfit for performing intubation of the larynx consists of five different-sized tubes, an instrument to insert them and another to extract with. A mouth-gag and gauge are also included. These instruments enclosed in a case are now sold for $35.00, and with such a set dyspnœa from laryngeal stenosis may be relieved in a surprisingly quick manner. Nevertheless, a large number of cases upon whom intubation is practiced die, showing that even the entrance of air to the lungs will not save the patient's life in many cases, and death finally occurs from such complications as pneumonia, sepsis, bronchitis or nephritis.

Some time ago Dr. Alfred S. Purdy, of this city, reported, according to law, to the board of health that one of his patients was suffering from small-pox. The health board thereupon had her removed to the small-pox hospital, where the physicians in charge denied that she had small-pox. Some time later she brought a suit for damages against Dr. Purdy, claiming that she had been improperly sent to the hospital and that her business had suffered in consequence. Dr. Purdy had certainly only obeyed the law in reporting what he considered a contagious disease, and he still believed that his patient had had small-pox; but, strange to say, a verdict was given in favor of the patient. The County Medical

Society then called a special meeting to ascertain if physicians were to be fined $500 for reporting cases of contagious diseases, and it was decided to ask all members of the profession in the city to contribute money to defend Dr. Purdy in another trial. This was done and as a result the general term have reversed the first decision and exonerated the doctor from all blame.

A few years ago it was not always an easy matter to obtain the services of a physician at night. Many doctors refused to make any-night calls and others would only go out for their regular patients. Strangers and others in the city were thus often obliged to go from office to office in search of medical aid. This state of things led to the organization of the "Night Medical Service," a charity which provides physicians at night for those who cannot pay a doctor, and yet, strange to say, it is seldom taken advantage of. Many of the people here are not aware that such a service exists and the calls only average about one a day. No doubt our mortality would be somewhat lower if the poor only knew that free doctors could be had at night. A physician, under these circumstances, may always be obtained by simply notifying the police. For this the city will pay a doctor three dollars a visit if the patient is unable to furnish the money. Blanks are on hand at all the police stations, which are filled out and given to the doctor. These papers are cashed by the board of health. The city is generally a little slow in paying, but the money is considered sure. Any doctor may join the service and many of our best, and especially the younger ones, are registered in the police stations, but the number of calls are still very few. Doctors expect to be paid at every visit among the poorer classes of people and their patients know it. Then again there are many doctors who at night insist on being paid before going out, as experience has taught them that this is the only way in which they can get their money.

A new hospital for the treatment of phthisis and incurable diseases is to be erected during the present year at 144th street and St. Ann's avenue. In 1882 a benevolent gentleman furnished buildings and property on East 109th street, and under the charge of the Sisters of the Poor, of St. Francis, an institution was started there for the cure of consumptives and called St. Joseph's Hospital; but the limited accommodations here of only fifty beds have proved to the management the necessity of having more room for the treatment of that numerous and ever-increasing class of patients who are incurable. The new hospital will have a capacity for about 250 beds and will cost $150,000. It will be managed by the same order of sisters who care for the sick at St. Francis Hospital, in East 5th street, and will be free to all proper patients who apply for aid.

At last accounts the hospital Sunday fund had reached $24,-843.19 and the prospects are good for a larger collection than ever before. A. F.

*January, 1887.*

---

BATTEY'S OPERATION IN PARIS.—The Paris correspondent of the *Lancet* says: "After meeting with considerable opposition here when first made known, Battey's operation is gradually coming into favor, and, though we cannot as yet compete in surgical activity with Liverpool, a number of speakers were able to give their experience of the operation at the recent discussion at the *Societie de chirurgie.* M. Terrier mentioned the case of a woman who, manifestly hysterical, suffered from intolerable pains in the ovaries at the menstrual periods. No relief being obtained by ordinary means, and life being a misery, these organs were removed last February. The right ovary caused no difficulty, but the left was so bound down by adhesions that it could only be extracted piecemeal. Since the operation there has been but insignificant malaise at the catamenial periods, which are perfectly normal. The hysterical condition, although it remains, appears amended; there are no longer any abdominal pains, and the general health is good. This is is the second success of the kind obtained by M. Terrier. M. Lucas-Championniere has also performed the operation in two cases; one of his patients died three days after the operation; the other was relieved of her symptoms, and at the same time cured of bad temper. Before the operation she easily became angry at trifles, whereas she is now quite calm. M. Monod said that he had also been successful in two instances, and M. Pozzi was able to indorse the treatment by one operation."—*New York Medical Journal.*

